# Supplementary material for: Potent antibody lineage against malaria transmission elicited by human vaccination with Pfs25
Source: Nat Commun. 2019 Sep 24;10:4328. doi: 10.1038/s41467-019-11980-6 (PMC6760140; doi:10.1038/s41467-019-11980-6)
Supplement: Supplementary file 1 — Supplementary Information [file 41467_2019_11980_MOESM1_ESM.pdf]

Supplementary information for

Potent antibody lineage against malaria transmission elicited by human vaccination with Pfs25

Authors

Brandon McLeod[1,2], Kazutoyo Miura[3], Stephen W. Scally[1], Alexandre Bosch[1], Ngan Nguyen[4], Hanjun Shin[4], Dongkyoon Kim[4], Wayne Volkmuth[4], Sebastian Rämisch[5], Jessica A. Chichester[6], Stephen Streatfield[7], Colleen Woods[8], William R. Schief[5], Daniel Emerling[4], C. Richter King[8], Jean-Philippe Julien*[1,2,9]

Affiliations

[1]Program in Molecular Medicine, The Hospital for Sick Children Research Institute, 686 Bay St, Toronto, Ontario, M5G 0A4, Canada.

[2]Department of Biochemistry, University of Toronto, 1 King's College Circle, Toronto, Ontario, M5S 1A8, Canada.

[3]Laboratory of Malaria and Vector Research, National Institute of Allergy and Infectious Diseases, National Institutes of Health, 12735 Twinbrook Parkway, Rockville, Maryland, 20852, USA.

[4]Atreca, 500 Saginaw Drive, Redwood City, California, 94063-4750, USA.

[5]Department of Immunology and Microbial Science, The Scripps Research Institute, La Jolla, California 92037, USA.

[6]Gene Therapy Program & Orphan Disease Center, Perelman School of Medicine, The University of Pennsylvania, Philadelphia, Pennsylvania, 19104, USA.

[7]Fraunhofer USA Center for Molecular Biotechnology CMB, 9 Innovation Way, Newark, Delaware 19711, USA.

[8]PATH's Malaria Vaccine Initiative, 455 Massachusetts Avenue NW Suite 1000, Washington, DC 20001, USA.

[9]Department of Immunology, University of Toronto, 1 King's College Circle, Toronto, Ontario, M5S 1A8, Canada.

*Corresponding author: jean-philippe.julien@sickkids.ca

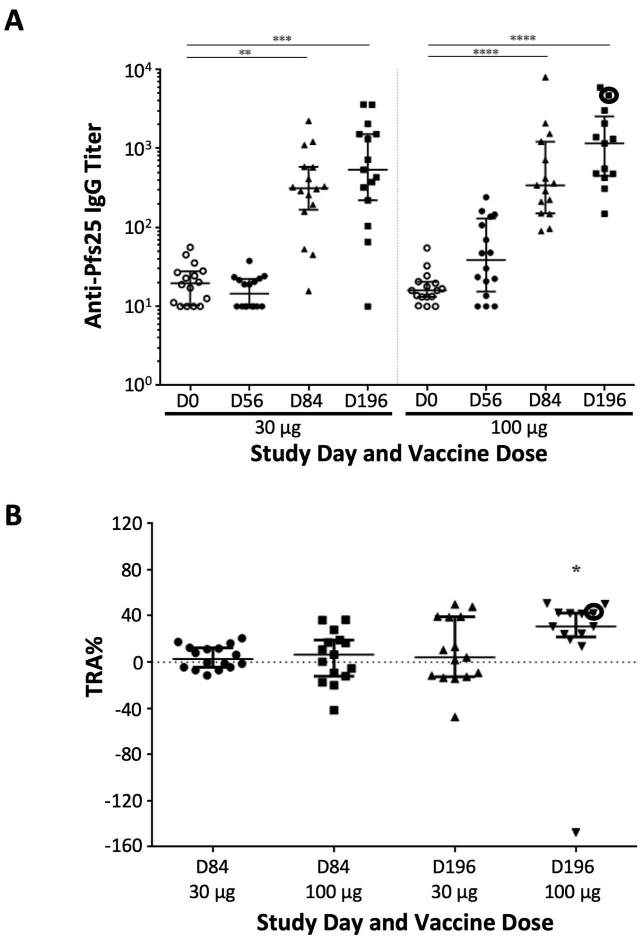

Supplementary Figure 1. Antibody titers and sera potency from human volunteers in a Phase 1 clinical trial[1]. A) Anti-Pfs25 IgG responses in serum samples collected from the 30 and 100 µg dose groups. Data are shown as median with interquartile range. **: $p < 0.01$, ***: $p < 0.001$, and ****: $p < 0.0001$ when compared to pre-immune data using the Friedman test followed by the Dunn's multiple comparison test. The circled data point indicates the serum sample from study day 196 (1 month post 3rd vaccination) corresponding to the individual from which antibody 2544 was recovered. **B)** Results of SMFA on sera collected from the 30 and 100 µg dose groups on Study Days 84 (1-month post 2nd dose) and 196 (1-month post 3rd dose). Purified IgG was tested at 3.75 mg/mL in the assay. Data are shown as median with interquartile range. Results are shown as the best estimates from 2 or 3 independent SMFA. *: $p < 0.05$ by the binomial test. The circled data point indicates the serum sample from study day 196 (1 month post 3rd vaccination) corresponding to the individual from which antibody 2544 was recovered.

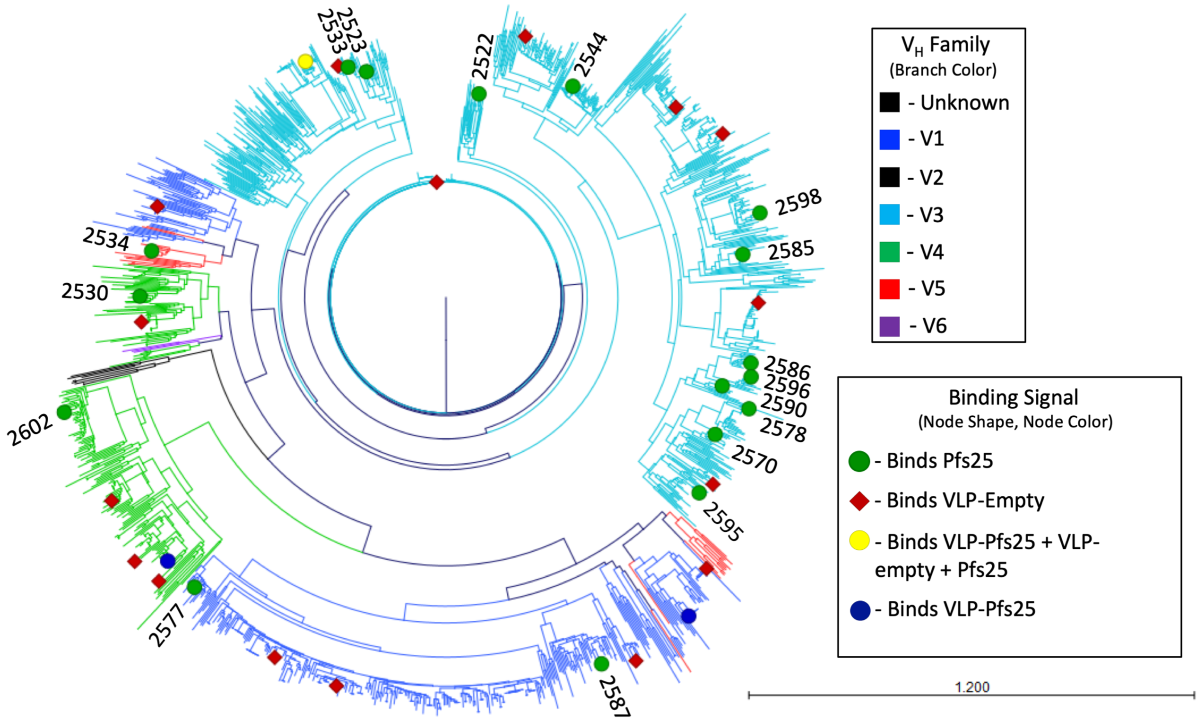

Supplementary Figure 2. Phylogram representation of plasmablast IgG repertoire responses to Pfs25 vaccination. Natively paired H- and L-chain, full-length, variable region sequences were combined into one phylogram based on full variable region H- and L-chain amino acid sequence similarity among 1,484 plasmablast IgG sequences. Between pairs of nodes, sum of radial distances is proportional to the Kimura distance as indicated by the scale bar. Branch color denotes the heavy chain V-gene assignment of the sequence at the corresponding node. Thirty-eight antibody sequences were recombinantly expressed and tested in three different ELISA assays for binding to vaccine components: AlMV Coat Protein (VLP-Empty), soluble Pfs25, and the VLP-Pfs25 vaccine[1,2]. Antibodies were observed to either be positive against both VLP-empty and Pfs25-VLP vaccine (red diamonds, n=16), positive against both Pfs25 and Pfs25-VLP vaccine (green circles, n=17), positive against Pfs25, Pfs25-VLP vaccine, and empty VLP (yellow circle, n=1), and positive against only the Pfs25-VLP vaccine (blue circles, n=2).

A

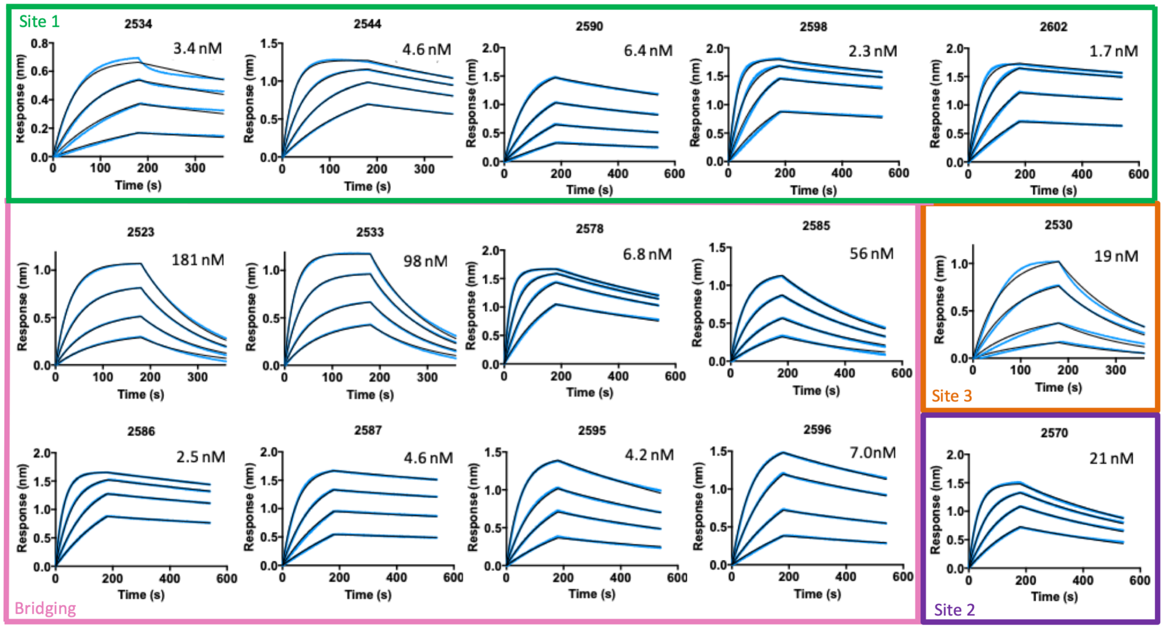

B

Fab	K_D (nM)	K_{on} (1/Ms)	K_{off} (1/s)	TRA% (100 µg/mL)
2523	181 ± 18	$4.1 \times 10^4 \pm 7.2 \times 10^3$	$7.2 \times 10^{-3} \pm 5.3 \times 10^{-4}$	70.9
2530	19.2 ± 1.9	$3.2 \times 10^5 \pm 3.8 \times 10^4$	$6.2 \times 10^{-3} \pm 1.8 \times 10^{-4}$	88.3
2533	98.8 ± 1.2	$7.7 \times 10^4 \pm 3.8 \times 10^3$	$7.6 \times 10^{-3} \pm 2.8 \times 10^{-4}$	50.5
2534	3.4 ± 0.2	$3.4 \times 10^5 \pm 1.8 \times 10^4$	$7.8 \times 10^{-4} \pm 6.6 \times 10^{-4}$	87.4
2544	4.6 ± 1.2	$1.6 \times 10^5 \pm 1.8 \times 10^4$	$7.5 \times 10^{-4} \pm 2.8 \times 10^{-4}$	100
2570	21 ± 7.9	$1.2 \times 10^5 \pm 3.7 \times 10^3$	$2.5 \times 10^{-3} \pm 8.7 \times 10^{-4}$	31.1
2578	6.8 ± 1.4	$1.8 \times 10^5 \pm 5.2 \times 10^3$	$1.2 \times 10^{-3} \pm 2.7 \times 10^{-4}$	82.5
2585	56 ± 17	$2.9 \times 10^4 \pm 2.5 \times 10^4$	$2.2 \times 10^{-3} \pm 4.2 \times 10^{-4}$	79.6
2586	2.5 ± 0.2	$1.5 \times 10^5 \pm 4.6 \times 10^3$	$3.7 \times 10^{-4} \pm 1.8 \times 10^{-5}$	92.2
2587	4.6 ± 2.5	$1.5 \times 10^5 \pm 5.0 \times 10^3$	$7.0 \times 10^{-4} \pm 3.6 \times 10^{-4}$	84.5
2590	6.4 ± 0.2	$9.1 \times 10^4 \pm 1.1 \times 10^4$	$5.9 \times 10^{-4} \pm 5.6 \times 10^{-5}$	77.7
2595	4.2 ± 2.3	$1.4 \times 10^5 \pm 8.1 \times 10^3$	$6.1 \times 10^{-4} \pm 3.7 \times 10^{-4}$	86.4
2596	7.0 ± 0.5	$1.3 \times 10^5 \pm 1.2 \times 10^4$	$9.3 \times 10^{-4} \pm 1.5 \times 10^{-4}$	68.9
2598	2.3 ± 0.2	$1.9 \times 10^5 \pm 4.8 \times 10^4$	$4.4 \times 10^{-4} \pm 7.7 \times 10^{-5}$	81.6
2602	1.7 ± 0.3	$2.2 \times 10^5 \pm 1.3 \times 10^4$	$3.9 \times 10^{-4} \pm 9.2 \times 10^{-5}$	83.5

Supplementary Figure 3. Kinetics of human Fabs binding to Pfs25 and mAb SMFA. A) Representative kinetic curves and epitope bins for the 15 human Fabs binding to Pfs25. Binding data was obtained by BLI, by immobilizing Pfs25 on Ni-NTA biosensors and associating with serially diluted Fabs (maximal concentration of 500 nM). The K_D from the representative measurement is indicated in each panel. **B)** Summary table of binding kinetics and SMFA data. All BLI data was collected as described in **A**). Fabs were assessed at least in duplicate experiments, with the associated error (standard deviation) beside the measurements. SMFA is for the mAb.

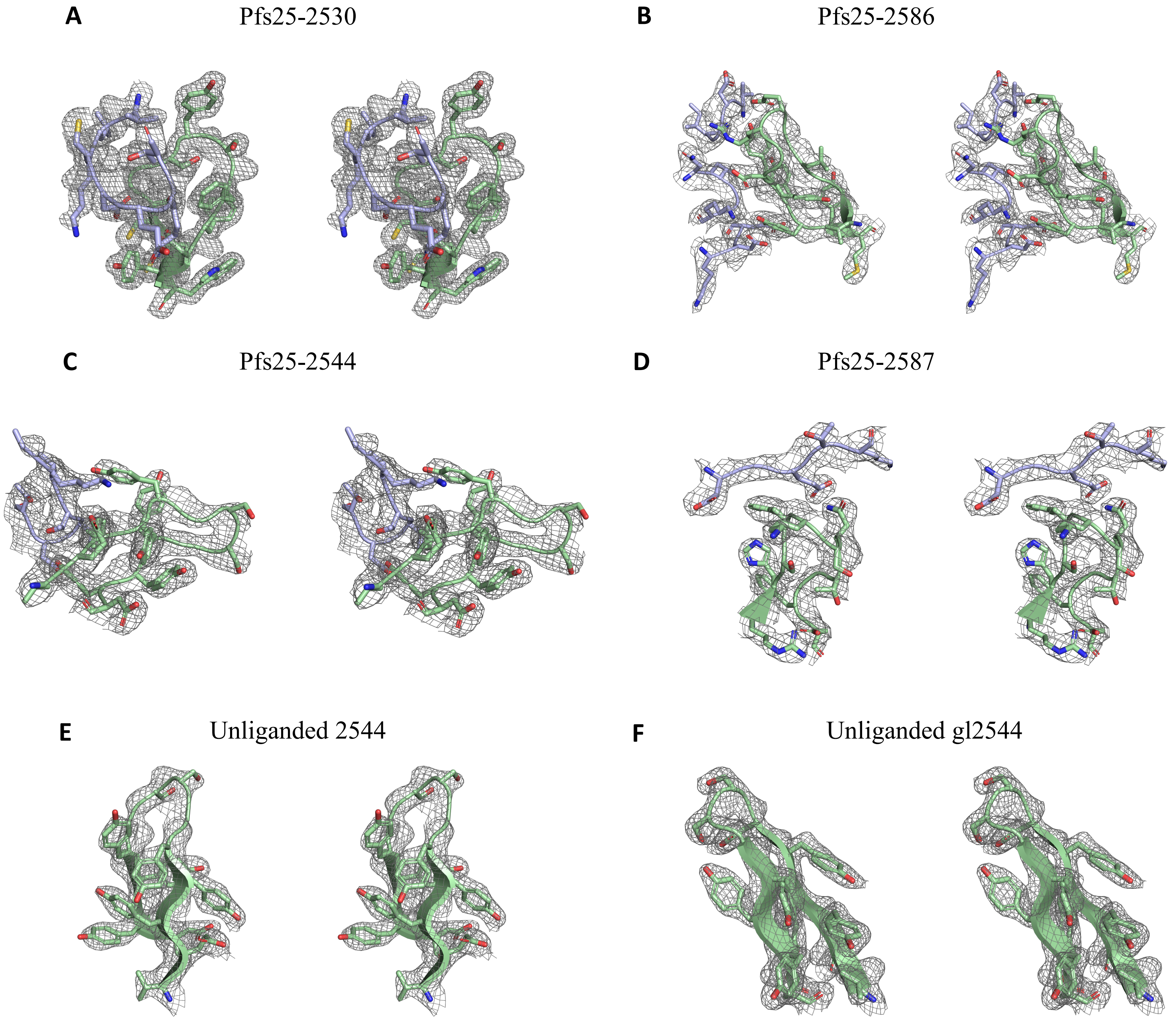

Supplementary Figure 4. Stereo view of anti-Pfs25 antibody HCDR3 and interacting Pfs25 residues. Stereo view of the 2mFo-DFc electron density map contoured at 1.0 σ for the following crystal structures: **A)** Pfs25-2530, **B)** Pfs25-2586, **C)** Pfs25-2544, **D)** Pfs25-2587, **E)** Unliganded 2544 and **F)** Unliganded Germline 2544. Pfs25 is depicted in light blue, all Fabs in pale green, and the electron density in grey mesh.

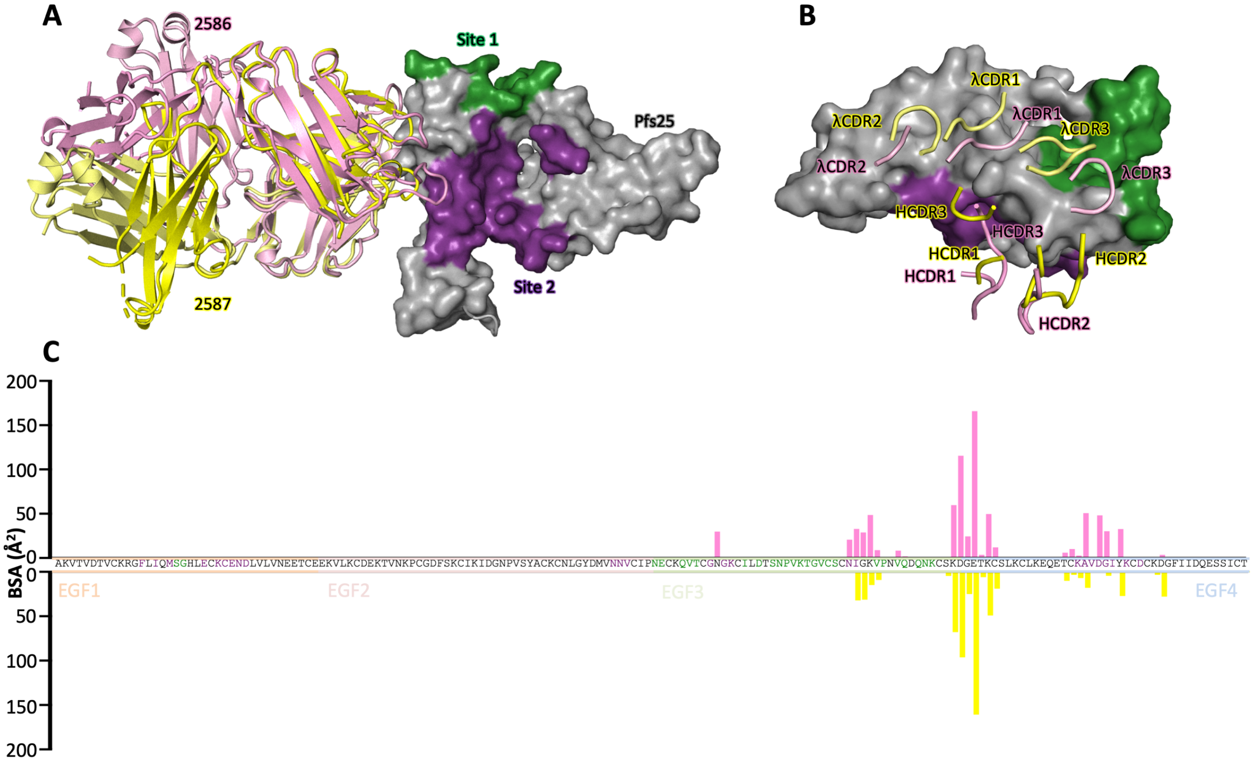

Supplementary Figure 5. Comparison of 2586 and 2587 interactions with Pfs25. A) Superposition of Fabs 2586 (pink) and 2587 (yellow) aligned on Pfs25 (Cα). Fabs are represented as cartoons, and Pfs25 (grey) is represented as surface, with the canonical Site 1 and Site 2 epitopes denoted by green and purple, respectively. **B)** Top-down view of 2586 and 2587 CDR interactions with Pfs25. Proteins are colored as defined in **A)** and CDRs are labeled. **C)** Epitope comparison between 2586 and 2587. Pfs25 sequence is written in black, with green and purple designating the canonical Site 1 and 2 epitopes, respectively. Pfs25 is further delineated into its four EGF domains (EGF1, wheat; EGF2, pale pink; EGF3, pale green; EGF4, pale blue). Buried surface area (BSA) by Pfs25 residue is represented by pink and yellow bars for 2586 and 2587, respectively.

A

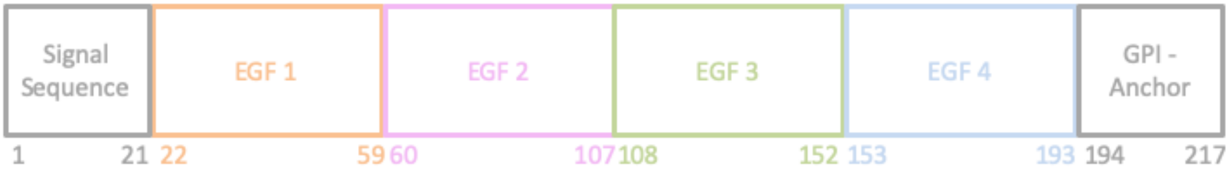

B

mAb	EGF Domain Contacted
2530	EGF1, EGF2
2544	EGF1, EGF3, EGF4
2586	EGF3, EGF4
2587	EGF3, EGF4
1190	EGF2, EGF3
1245	EGF1, EGF2, EGF3, EGF4
1260	EGF1, EGF2, EGF3, EGF4
1262	EFG3
1269	EGF1, EGF3
1276	EFG3

Supplementary Figure 6. Schematic of Pfs25 EGF domains and mAb interactions. A) Schematic of Pfs25, separated by signal sequence, EGF domains, and GPI-anchor. Domains are indicated by boxes, and residue numbers corresponding to each domain are reported below the boxes. Residue numbers are according to Uniprot accession P13829. **B)** Table summarizing crystalized humanized mouse[3] and human mAbs with the EGF domains the mAb contacts.

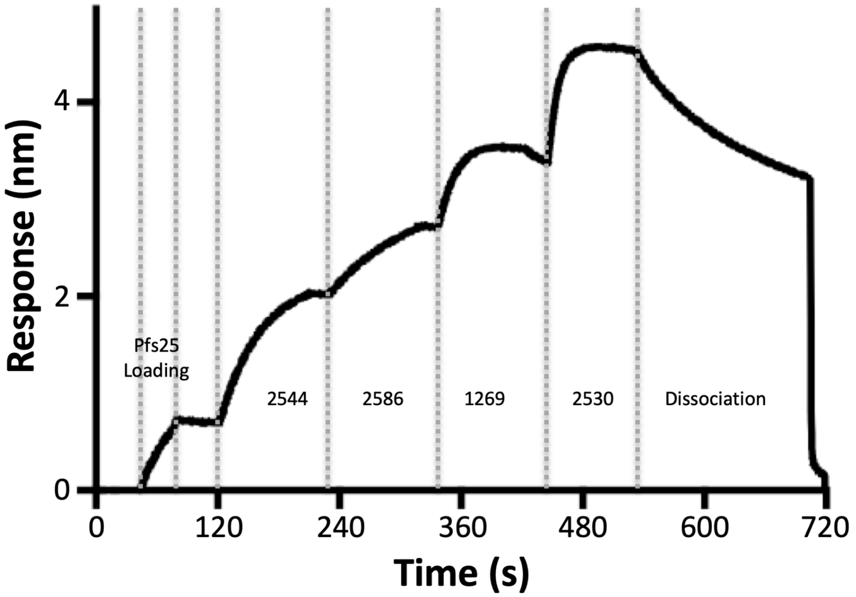

Supplementary Figure 7. Sequential binding of four Fabs on Pfs25. BLI was conducted with Pfs25 immobilized on a Ni-NTA biosensor, and associated into subsequent Fabs of known, non-overlapping epitopes: 2544 (Site 1b), 2586 (bridging), 1269 (Site 1a), and 2530 (Site 3). A 10 s baseline was established between each new association. Each increase in response indicates Fab binding to Pfs25.

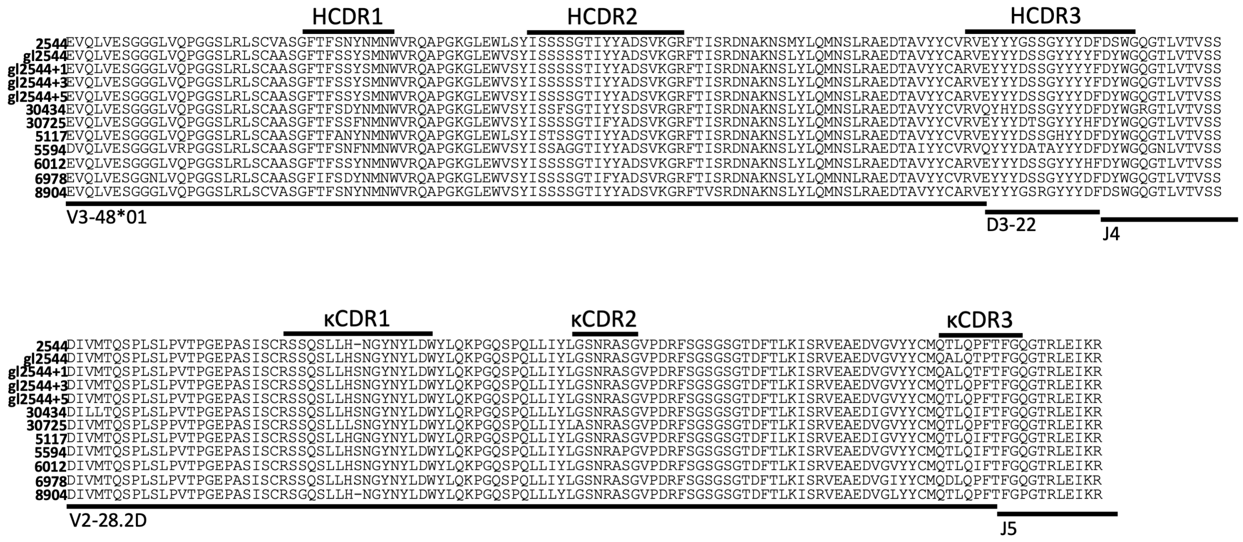

Supplementary Figure 8. Variable heavy (V_H) and kappa (V_K) sequences for 2544, germline 2544, and minimally-mutated germline forms, in addition to the 7 lineage members that were expressed and analyzed. CDRs for both chains are labelled above the sequences, and predicted VDJ-encoding regions are denoted below the sequences.

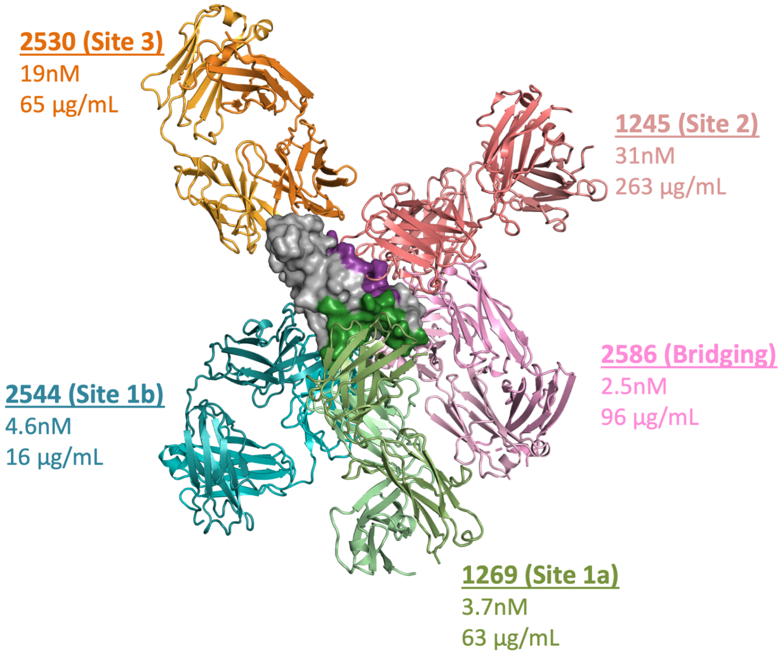

Supplementary Figure 9. Structures of representative Fabs from each major epitope bin superposed on Pfs25. Site 1a (1269, green)[3], Site 1b (2544, teal), Bridging (2586, pink), Site 2 (1245, salmon), and Site 3 (2530, orange)[3], are labelled, and their associated affinity to recombinant Pfs25 and IC_{80} in SMFA are listed.

Supplementary Table 1. Characterization of gl2544 mutated mAbs. No binding (NB). Unable to fit (UTF). Transmission reducing activity (TRA).

mAb	K_D (M)	K_{on} (1/Ms)	K_{off} (1/s)	TRA (%) @ 375 µg/mL
gl2544	NB	NB	NB	0
gl2544+1	UTF	UTF	UTF	0
gl2544+3	$2.01\ 10^{-6} \pm 3.53 \times 10^{-8}$ M	$8.17 \times 10^{4} \pm 2.12 \times 10^{2}$	$1.65 \times 10^{-1} \pm 2.83 \times 10^{-3}$	0
gl2544+5	$5.11\ 10^{-8} \pm 9.19 \times 10^{-10}$	$6.72 \times 10^{4} \pm 1.91 \times 10^{3}$	$3.43 \times 10^{-3} \pm 3.54 \times 10^{-5}$	77.9

Supplementary References:

1. Chichester, J. A. *et al.* Safety and immunogenicity of a plant-produced Pfs25 virus-like particle as a transmission blocking vaccine against malaria: A Phase 1 dose-escalation study in healthy adults. *Vaccine* **36**, 5865–5871 (2018).
2. Jones, R. M. *et al.* A novel plant-produced Pfs25 fusion subunit vaccine induces long-lasting transmission blocking antibody responses. *Hum Vaccines Immunother.* **11**, 124–132 (2015).
3. Scally, S. W. *et al.* Molecular definition of multiple sites of antibody inhibition of malaria transmission-blocking vaccine antigen Pfs25. *Nat. Commun.* **8**, 1568 (2017).
