# Supplementary material for: Potent antibody lineage against malaria transmission elicited by human vaccination with Pfs25
Source: Nat Commun. 2019 Sep 24;10:4328. doi: 10.1038/s41467-019-11980-6 (PMC6760140; doi:10.1038/s41467-019-11980-6)
Supplement: Supplementary file 3 — Reporting Summary [file 41467_2019_11980_MOESM3_ESM.pdf]

nature research

Corresponding author(s): Jean-Philippe Julien

Last updated by author(s): Aug 13, 2019

Reporting Summary

Nature Research wishes to improve the reproducibility of the work that we publish. This form provides structure for consistency and transparency in reporting. For further information on Nature Research policies, see Authors & Referees and the Editorial Policy Checklist.

Statistics

For all statistical analyses, confirm that the following items are present in the figure legend, table legend, main text, or Methods section.

n/a	Confirmed	
☐	☒	The exact sample size (n) for each experimental group/condition, given as a discrete number and unit of measurement
☐	☒	A statement on whether measurements were taken from distinct samples or whether the same sample was measured repeatedly
☐	☒	The statistical test(s) used AND whether they are one- or two-sided<br>*Only common tests should be described solely by name; describe more complex techniques in the Methods section.*
☒	☐	A description of all covariates tested
☐	☒	A description of any assumptions or corrections, such as tests of normality and adjustment for multiple comparisons
☐	☒	A full description of the statistical parameters including central tendency (e.g. means) or other basic estimates (e.g. regression coefficient) AND variation (e.g. standard deviation) or associated estimates of uncertainty (e.g. confidence intervals)
☐	☒	For null hypothesis testing, the test statistic (e.g. F, t, r) with confidence intervals, effect sizes, degrees of freedom and P value noted<br>*Give P values as exact values whenever suitable.*
☒	☐	For Bayesian analysis, information on the choice of priors and Markov chain Monte Carlo settings
☒	☐	For hierarchical and complex designs, identification of the appropriate level for tests and full reporting of outcomes
☒	☐	Estimates of effect sizes (e.g. Cohen's d, Pearson's r), indicating how they were calculated

Our web collection on statistics for biologists contains articles on many of the points above.

Software and code

Policy information about availability of computer code

Data collection	X-ray diffraction data were collected at the 08ID-1 beamline at the Canadian Light Source (CLS), processed, and scaled using XDS.
Data analysis	Structures were determined by molecular replacement using Phaser and Pfs25 as a search model. Refinement of the structures was carried out using phenix.refine and model building iterations in Coot. SBGrid was used to access all crystallography software. Molecular modeling of 2544 lineage: All models were generated from the 2544-Pfs25 complex crystal structure. This structure was prepared using QuickPrep in MOE, and models were generated through MOE's residue scanning function. Predicted affinities between antibodies and the antigen were determined by model generation using MOE's scoring algorithms. Resulting homology models were further analyzed using the PDBePISA server.

For manuscripts utilizing custom algorithms or software that are central to the research but not yet described in published literature, software must be made available to editors/reviewers. We strongly encourage code deposition in a community repository (e.g. GitHub). See the Nature Research guidelines for submitting code & software for further information.

Data

Policy information about availability of data

All manuscripts must include a data availability statement. This statement should provide the following information, where applicable:

- Accession codes, unique identifiers, or web links for publicly available datasets
- A list of figures that have associated raw data
- A description of any restrictions on data availability

Structural data has been deposited to the Protein Data Bank (PDB) under accession codes: 6PHB, 6PHC, 6PHD, 6PHF, 6PHG, and 6PHH.

Field-specific reporting

Please select the one below that is the best fit for your research. If you are not sure, read the appropriate sections before making your selection.

☒ Life sciences ☐ Behavioural & social sciences ☐ Ecological, evolutionary & environmental sciences

For a reference copy of the document with all sections, see nature.com/documents/nr-reporting-summary-flat.pdf

Life sciences study design

All studies must disclose on these points even when the disclosure is negative.

Sample size	For SMFA: The mosquitoes were maintained for 8 days and then dissected to count the number of oocysts per midgut in 20 mosquitoes. The best estimate of %TRA, the 95% CI, and significance of inhibition from single or multiple feeds were calculated as previously described using a zero-inflated negative binomial model. The Bliss independence model i.e. assuming mAbs act independently was used to determine the theoretical additive effect.
Data exclusions	No data were excluded from the analyses.
Replication	All attempts at replication were successful, with given confidence intervals.
Randomization	No randomization was necessary for our biophysical and structural studies.
Blinding	Blinding was not necessary in our biophysical and structural studies.

Reporting for specific materials, systems and methods

We require information from authors about some types of materials, experimental systems and methods used in many studies. Here, indicate whether each material, system or method listed is relevant to your study. If you are not sure if a list item applies to your research, read the appropriate section before selecting a response.

Materials & experimental systems

n/a	Involved in the study	
☒	☐	Antibodies
☒	☐	Eukaryotic cell lines
☒	☐	Palaeontology
☒	☐	Animals and other organisms
☐	☒	Human research participants
☒	☐	Clinical data

Methods

n/a	Involved in the study	
☒	☐	ChIP-seq
☒	☐	Flow cytometry
☒	☐	MRI-based neuroimaging

Human research participants

Policy information about studies involving human research participants

Population characteristics	Our analysis focused on the B cell response of one participant who achieved high transmission-reduction activity (76.9% at 15 mg/ml) in the phase I clinical trial study registered at www.ClinicalTrials.gov under reference identifier NCT02013687.
Recruitment	Our study performed B cell analyses on human samples from a previously-published phase I clinical trial study registered at www.ClinicalTrials.gov under reference identifier NCT02013687.
Ethics oversight	The study protocol was approved by the Western Institutional Review Board, and all participants provided written informed consent.

Note that full information on the approval of the study protocol must also be provided in the manuscript.
